# Childhood Obesity Prevention and General Practice: A Mapping Review of Australian Clinical Resources

**DOI:** 10.1002/hpja.70006

**Published:** 2025-04-09

**Authors:** Michelle Gooey, Helen Skouteris, Kellie West, Peter Bragge, Elizabeth Sturgiss

**Affiliations:** ^1^ Health and Social Care Unit, School of Public Health and Preventive Medicine Monash University Victoria Australia; ^2^ Warwick Business School University of Warwick Coventry UK; ^3^ School of Primary and Allied Health Care Monash University Victoria Australia; ^4^ BehaviourWorks Australia, Monash Sustainable Development Institute Monash University Victoria Australia

**Keywords:** general practice, health promotion, paediatric obesity, preventive medicine, primary prevention

## Abstract

**Issue Addressed:**

Preventing childhood obesity is a health promotion priority in Australia, and general practitioners (GP) play an important role through the provision of preventive healthcare. We identified and characterised existing Australian clinical resources which could support childhood obesity prevention in general practices to better understand resource availability and identify gaps to facilitate the planning of possible future interventions.

**Methods:**

A mapping review was undertaken to find relevant clinical resources that focus on growth monitoring and/or promoting healthy behaviours relevant to children with a healthy weight. In this review, a ‘clinical resource’ is a resource for use in a patient consultation. All resources were independently assessed by two practising GP investigators for clinical use suitability. Additionally, the Patient Education Materials Assessment Tool (PEMAT) or an author‐adapted Royal Australian College of General Practitioners tool was used to assess each resource as appropriate.

**Results:**

One hundred and twenty resources were included. The target audience was children and/or their families for 114 resources, and GPs for six. GP involvement was found in the development of one resource. Overall, mean PEMAT scores indicated that many patient materials were understandable but poorly actionable.

**Conclusions:**

There are many existing Australian resources relevant to childhood obesity prevention in general practice. Most are directed towards children and their families; however, quality assessment indicated improvements are needed to support action.

**So What?:**

Partnering with GPs, children, and their families is an important next step to ensure that health‐promoting clinical resources available for general practice are optimised for use.

## 1 Introduction

Preventing childhood obesity is a health promotion priority in Australia [1] and around the world [2]. In Australia, promoting health through the provision of preventive care is an important part of a general practitioner's (GP) practice [3] through activities such as growth monitoring and promotion of healthy behaviours as outlined by the Royal Australian College of General Practitioners Guidelines for Preventive Activities (or ‘Red Book’) [3]. These activities are also consistent with international guidelines for the prevention of childhood obesity [4].

The Royal Australian College of General Practitioners (RACGP) Red Book recommends regular growth monitoring for all children, including the calculation of Body Mass Index in children from 2 years of age [3]. GPs are also encouraged to discuss healthy behaviours such as diet, including breastfeeding, physical activity, sleep, and sedentary behaviour with their paediatric patients and their families, in an age‐specific manner [3]. Although the Red Book only includes sleep and sedentary behaviour recommendations for some paediatric age groups [3], the Australian 24‐h movement guidelines provide sleep, sedentary behaviour, and physical activity recommendations for children of all ages [5, 6].

The RACGP specifically recommends the use of patient education materials to support preventive care delivery in a clinical consultation [7]. This is consistent with findings that the use of supportive written and audiovisual materials in conjunction with direct patient education and behaviour change techniques has been associated with beneficial preventive care outcomes [8]. More specific to childhood obesity, patient education materials have been recognised as an important adjunct to support healthy behaviour promotion in the primary care setting [9].

Clinician‐facing resources can also be used to support guideline implementation and interpretation—for example, posters in consultation rooms, practical guideline‐related tools, and decision support tools [9, 10]. However, consistent with findings internationally [11], it can be challenging for Australian GPs to locate appropriate resources that support obesity prevention activities in their day‐to‐day practice [12]. Furthermore, this has been a long‐standing issue in paediatric preventive care more broadly [13].

Thus, we sought to better understand the breadth and characteristics of resources available to support childhood obesity prevention in Australian general practices, as well as to identify gaps to facilitate the planning of possible future interventions to enhance this practice. The objective of this study was to identify and characterise existing Australian clinical resources that could support obesity prevention in children with a healthy weight in general practice.

## 2 Methods

A mapping review was undertaken to describe key characteristics of relevant resources. Mapping reviews identify and categorise existing literature to improve understanding of the current situation relating to a particular topic [14, 15], for example, to determine research priorities and funding [16]. Although similar to a scoping review, some differentiating features include engagement with stakeholders and graphical representation of findings [16]. Additionally, there is less focus on *content* as significant data extraction is not done [14].

### 2.1 Key Eligibility Criteria

For the purposes of this review, a ‘clinical resource’ was defined as a resource for use *in the context of a patient consultation*. Examples include a quick reference guide for clinicians or information suitable to be shared with patients and/or their families (e.g., patient handouts, posters for display). Tools such as webinars and clinical practice guidelines, which are not usually used in the context of a patient consultation, were excluded.

Key inclusion criteria were:

- Australian resources that focus on growth monitoring and/or promoting healthy behaviours (healthy diet including breastfeeding, sleep, physical activity and sedentary behaviour)
- Relevant to children with a healthy weight up to the age of 17 years old
- Published by a government organisation, professional body, or authoritative paediatric healthcare organisation (e.g., specialist tertiary paediatric hospital) or funded by one of these organisations.

Resources were excluded if they specifically related to children who are living with overweight, obesity or an existing health condition/illness. To identify resources applicable to a relatively broad patient population as found in general practice, resources with a narrow focus or that covered a particular issue (e.g., recipe provision or fussy eating) or for a particularly restricted age range of less than 12 months (e.g., for 3–6‐month‐old babies) were also excluded. Other key exclusion criteria were resources specifically intended for settings outside of general practice; not in English; expired or withdrawn from use; or required a password and/or specific account to access. When determining the scope of this study, an important consideration was that clinical resources suitable for children and/or their families should be readily accessible to all patients. Internet access and digital exclusion are significant issues in Australia, with almost 10% of the population considered to be ‘highly excluded’ in 2023 [17]. Specific issues include affordability stress (e.g., people living with a disability or in public housing) [17] and poor infrastructure impacting rural and remote areas (and exacerbated by other social determinants of health [18]). Therefore, resources with video content were excluded as streaming can be difficult for those with limited or no access to the internet, whereas most more static resources can be printed out and given to the patient if required.

### 2.2 Search Strategy

The search strategy focused on grey literature (see Supporting Information for additional details) and was carried out by MG. Thirty‐one Australian websites were reviewed (see Supporting Information for full list), focusing on state/territory or national government organisations, professional bodies, and authoritative paediatric healthcare organisations that are likely to produce resources relevant to obesity prevention in general practice. Other searches using web search engines Google Scholar and Duck Duck Go were carried out between March and July 2023. Additional resources were sought through professional networks.

### 2.3 Screening Process

There were two phases of screening. In phase one, one researcher (M.G.) carried out the literature search and identified potentially relevant materials. Materials were then screened for eligibility by MG based on the inclusion and exclusion criteria. All candidate materials were then reviewed independently in the second phase by E.S. and K.W. (a practicing GP and GP registrar respectively, henceforth referred to as GP investigators) in Covidence to assess suitability as a clinical resource in general practice, considering the question ‘Would I be able to use this resource in my clinical consultations?’. In this phase, agreement was required by both GP investigators to be included. Conflicts were resolved through discussion between E.S. and K.W. Although input regarding resource suitability from additional GPs would be optimal, logistical factors such as time and resource constraints precluded this. Whilst clearly not a representative sample of all Australian GPs, it is worth noting some diversity in the two screening GP investigators' clinical practice settings—they are based in different Australian states/territories, and ES practices in a capital city whilst KW practices in a regional setting.

### 2.4 Data Analysis and Quality Assessment

MG assessed whether the material was relevant primarily as a GP or child/family resource; E.S. and K.W. provided additional input where required. Key features of each resource (year published, type of organisation, originating jurisdiction, resource focus, GP involvement noted, specifically for Aboriginal and/or Torres Strait Islander people or culturally and linguistically diverse groups) were extracted into an Excel spreadsheet and analysed descriptively, including through the use of mapping diagrams. It was out of the scope of this review to verify the accuracy of information within included materials.

The quality assessment of each resource was undertaken by M.G. as follows:

#### 2.4.1 Clinical Resources for Children and/or Families

The Patient Education Materials Assessment Tool (PEMAT) [19] was used to assess patient materials (see Supporting Information for details). The PEMAT is a standardised tool for assessing patient material [19]. It is published by the United States Agency for Healthcare Research and Quality and is recommended by the Australian Commission on Safety and Quality in Health Care as a useful resource for preparing written information for consumers [20]. The PEMAT seeks to assess the Understandability and Actionability of the given material for a broad range of users [21]. Materials are said to be ‘understandable’ if diverse consumers can ‘process and explain key messages’ (p. 396) [21] and ‘actionable’ if diverse consumers can ‘identify what they can do based on the information presented’ (p. 396). The PEMAT assessment provides separate scores for Understandability and Actionability, which can facilitate comparison between different materials [19]. A score of 70% or more in either category indicates the resource is understandable or actionable as appropriate [21]. The PEMAT tool can be used for printable materials (PEMAT‐P; 24 items in total) and audiovisual materials (PEMAT‐A/V; 17 items in total) [19] with some overlap in items between the two tools. Web resources which could be converted to a PDF (e.g., for hard copy printing) were assessed with PEMAT‐P.

#### 2.4.2 Clinical Resources for GPs

We are not aware of a validated tool to assess the quality and suitability of clinical resources for general practitioners. Instead, indicators from a RACGP tool were used to assess whether a clinical resource is of sufficiently high standard for use in general practice [22] were adapted (see Supporting Information for details).

## 3 Results

**FIGURE 1:**
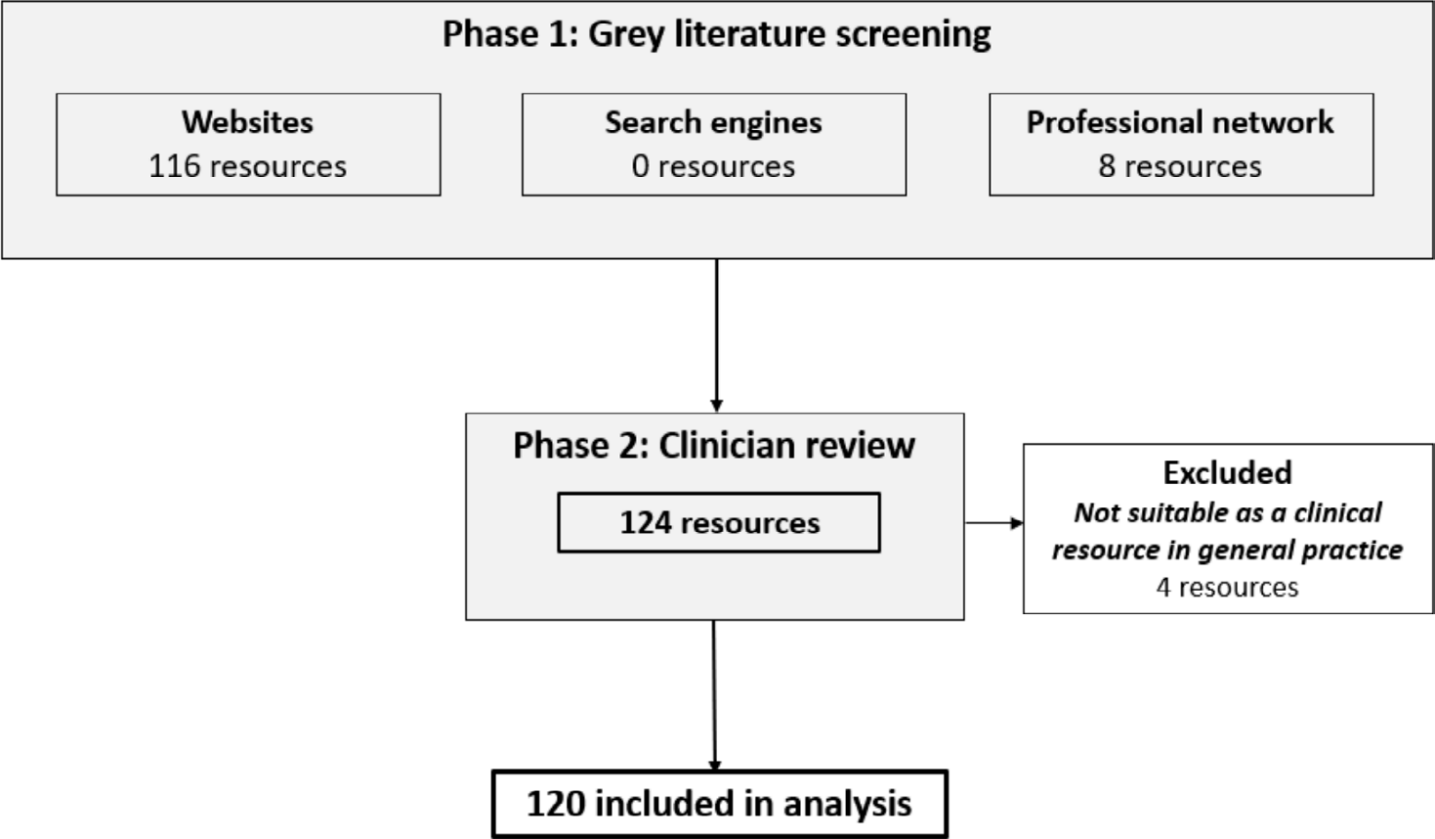
Search and screening overview.

Following the grey literature search, 116 resources plus 8 additional resources from professional network sources were included; no new resources from the web search engines were identified. Of these 124 resources, four resources were assessed not to be suitable as a clinical resource at the clinician review stage. Thus, 120 were included for analysis (see Figure 1).

### 3.1 Resource Characteristics

Of the 120 resources included, children and/or their families were assessed to be the primary audience for 114, and GPs for six (see Supporting Information for complete list). Overall (see Table 1), many resources were published relatively recently, although many did not state a publication date. Approximately three‐quarters of the resources were produced by a government organisation, and two‐thirds of resources were published by a state‐ or territory‐based entity. The majority focused on a single topic. Only one resource was found to have a GP involved in its development.

**TABLE 1:** Overview of characteristics of resources included in the mapping review.

	For children and/or families	For GPs	Total
Number of resources	114	6	120
Year published			
2018–2023	45	4	49
2013–2017	12	0	12
Before 2013	7	0	7
Not stated	50	2	52
Type of organisation			
Government	73	6	79
Healthcare provider	24	0	24
Professional body	2	0	2
Other	15	0	15
Originating jurisdiction			
National	28	2	30
State or territory	86	4	90
Resource focus			
Single topic	83	4	87
Combination of topics	31	2	33
Audience			
Aboriginal and/or Torres Strait Islander people	4	0	4
Culturally and linguistically diverse groups	6	0	6
GPs noted to be involved in resource development	1	0	1

## 4 Clinical Resources for Children and/or Families

### 4.1 Characteristics

**FIGURE 2:**
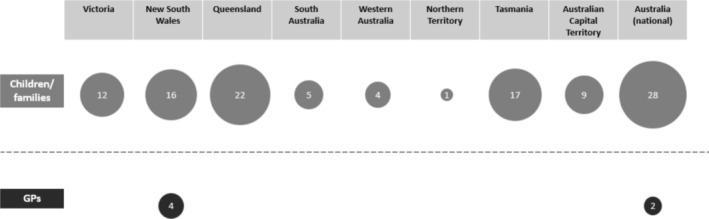
Number of resources by state/territory.

**FIGURE 3:**
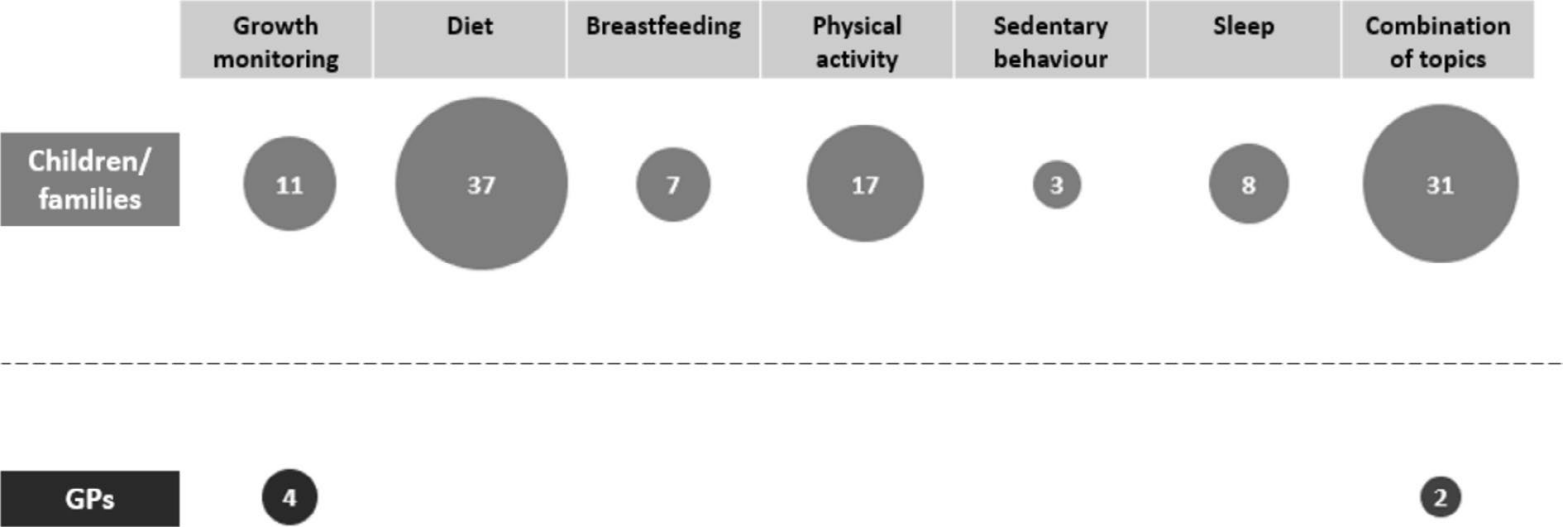
Number of resources by topic focus according to resource audience (children/families or GPs).

One hundred and fourteen relevant resources for children and/or families were identified. Many (45) of the resources were published recently (2018–2023) although a large number (50) did not identify a publication date. The majority were published by government organisations (73) such as government health departments or other affiliated organisations, such as state‐based health promotion agencies (e.g., Health and Wellbeing Queensland). Most originated from Australian states or territories (86), with Queensland (22) offering the most resources relevant to this review and Northern Territory offering the fewest (see Figure 2). There were 28 resources with a national scope. In terms of focus, the most common single topic was diet/nutrition (37; 44 including breastfeeding), followed by physical activity (17) (see Figure 3). Thirty‐one resources included a combination of topics.

Only one resource was found to have input from a GP. Four resources were assessed to be specifically for Aboriginal and/or Torres Strait Islander children and/or their families. An additional six were part of the Good Start suite of resources, designed for Maori and Pacific Islander children and their families.

### 4.2 Quality Assessment

**FIGURE 4:**
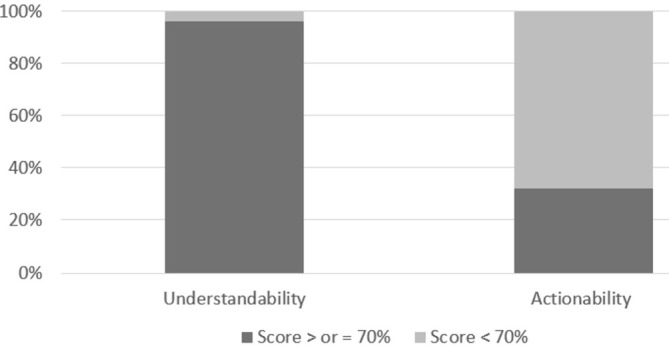
Understandability and actionability PEMAT scores of clinical resources for children and/or families.

One hundred and twelve resources were assessed using the PEMAT‐P and two resources using the PEMAT‐A/V. The mean Understandability score was 87% (range: 62%–100%) and 110 of the 114 (96%) resources scored 70% or more (see Figure 4). The mean Actionability score was 66% (range: 14%–100%) and 37 of the 114 resources (32%) had an Actionability score of 70% or more (see Figure 4). Thirty‐seven resources scored 70% or more in both categories and four resources scored less than 70% in both categories. Almost two thirds of resources (73) scored 70% or more for Understandability but less than 70% for Actionability.

In terms of individual items in the Understandability category, less than 50% of eligible resources were marked *agree* for ‘the material provides a summary’ (item 11) and ‘the material uses visual aids whenever they could make content more easily understood’ (item 15; PEMAT‐P only). In terms of Actionability items, less than 50% of eligible resources had *agree* marked for ‘the material provides a tangible tool (e.g., menu planners, checklists) whenever it could help the user take action’ (item 23; PEMAT‐P only), ‘the material explains how to use the charts, graphs, tables, or diagrams to take actions’ (item 25) and ‘the material uses visual aids whenever they could make it easier to act on the instructions’ (item 26; PEMAT‐P only). Of the scored items, one Actionability item ‘provides simple instructions or examples of how to perform calculations’ (item 24) was not found in any resource, however only five resources were eligible for this item.

## 5 Clinical Resources for GPs

### 5.1 Characteristics

Six resources for GPs were identified in this review. Most (4) were related to growth monitoring, all providing guidance on how to measure height, length, or weight. At least four resources were published in the last 5 years, although two did not have a publication date noted, and all were published by government organisations. Four resources originated from New South Wales (NSW). None of the resources assessed to be for GPs were found to have a GP involved in their development.

### 5.2 Quality Assessment

All six of the GP resources scored 50% on the panel of six adapted indicators. The indicators of ‘clearly written in an understandable form and the key recommendations are easily identifiable’ (indicator 1) and ‘an indication that the resource is likely to be evidence based’ (indicator 5) were consistently present on all GP resources. However, none of the resources included the indicators ‘the target users and the health care context addressed by the resource explicitly include general practitioners’ (indicator 2) or ‘the members of the resource development group have stated their conflicts of interest and the financial independence has documented and transparently managed’ (indicator 4).

## 6 Discussion

This is the first known attempt to systematically map existing Australian clinical resources relevant to childhood obesity prevention in general practice. We found 120 clinical resources relevant to general practice pertaining to growth monitoring and healthy behaviours in children. The majority were targeted at children and families and related to the promotion of healthy behaviours. In terms of topics, there were relatively few resources relating to sedentary behaviour compared with the other healthy behaviour topics. Only six resources were relevant to GPs, and of these, the most common focus was growth monitoring.

Written materials and visual aids can play an important role in patient education [23]. This review found many existing patient resources relevant to childhood obesity prevention with a variety of topics covered. Most of the resources were standalone resources on a single topic; the most common topics were diet/nutrition (including breastfeeding) and physical activity. Some jurisdictional‐based production of resources was noted, with both Queensland and NSW producing 20 or more resources each. This may reflect particular state‐based policy priorities—for example, the reduction of childhood overweight and obesity was previously a ‘Premier's Priority’ in NSW [24] and more recently, ‘Healthy weight’ has been designated as one of the focus areas for the Health and Wellbeing Queensland, the state's health promotion agency [25].

Although a large number of resources were identified in our study, this was primarily done through the time‐consuming process of manual searching websites which many GPs would not have the time to dedicate effort. It is therefore important that GPs are adequately supported to access information relevant to their practice and their patients. Recently, two online, publicly available, Australian resource repositories of relevance to this topic were launched however the ‘Go and Grow Toolbox’ [26] has a narrower focus containing only nutrition information pertaining to children under the age of five and the Health and Wellbeing Queensland ‘Clinical Toolkit’ supports the *management* (not prevention) of overweight and obesity [27]. Another option may be through resources for GPs, which have accompanying content intended to be shared with patients and supported by the same knowledge base. Examples found in this search include the Healthy Kids for Professionals website [28], which focuses on management of children above a healthy weight but includes some content relevant to prevention, and the Australian 24 h movement guidelines [29].

The provision of preventive care relevant to obesity has a clear place in general practice, as articulated by the RACGP [3] and general practice staff [30]. Furthermore, it aligns with the Ottawa Charter's goal to reorient health services towards the ‘pursuit of health’, rather than the treatment of illness [31]. However multiple barriers to obesity prevention impacts day‐to‐day practice. For example, the complexities associated with obesity prevention discussions, the challenges of providing preventive care in the current general practice system and the additional pressures that the COVID‐19 pandemic has put on general practice [30]. In the context of obesity prevention in general practice, clinical resources have been identified as a way to support action [12, 30]. However, finding ways to integrate clinical resource use into practice is also critical for implementation success. Relevant proposals from co‐ideation with Australian GPs included embedding links to relevant resources into clinical guidelines and creating an accessible repository of resources [12].

Of particular relevance, this review found only six resources suitable for GPs, and none were tailored specifically for general practice use. In part, this may be because traditional practitioner resources, such as education and training tools for professional development, were out of scope for this review. Nevertheless, the dearth of resources that are practically applicable for GPs within a patient consultation represents a missed opportunity to support preventive care in general practice.

Contextualisation is critical for successful implementation [32], and supportive tools should be tailored to address relevant implementation barriers and/or enablers. Future work should ensure that GPs and other relevant people, such as children and their families, contribute to the development of general practice resources through collaborative partnerships, as they are best placed to evaluate how well such resources address their needs and ensure practical applicability. Furthermore, the co‐design of consultation‐friendly resources is consistent with the recognition that the involvement of relevant stakeholders is an important step in health service design [33] and health promotion development [34].

The PEMAT assessment of patient‐facing resources [19] in this review indicated that whilst many resources had good understandability (a score of 70% or more [21]), this was often associated with low actionability (a score less than 70% [21]). This suggests that whilst these resources outlined the importance of addressing an issue effectively, they may lack practical guidance for promoting action amongst children and their families. The PEMAT was the first tool produced to measure the actionability of patient materials [21]; examples of relevant items include clear identification of at least one action, presenting actions in a stepwise manner, and providing tools such as checklists [19]. Other guidance for improving patient education material is readily available through a number of health agencies, including the Australian Commission on Safety and Quality in Health Care [20] and the United States' Office of Disease Prevention and Health Promotion [35].

Finally, the focus of general practice obesity prevention efforts in this review is on clinical consultation interactions. This is consistent with RACGP‐endorsed guideline recommendations [3] and the recognition that GPs are a trusted source of health information amongst Australian parents [36]. As suggested by general practice staff, Australian GPs and general practices could more broadly contribute to obesity prevention by considering the needs of their local community—for example, by tailoring services and initiating engagement outside of a clinical consultation [30]. Other suggestions include modelling behaviour as community leaders and advocating for policy change [37]. However, general practice action alone is unlikely to sufficiently address childhood obesity prevalence in Australia. Action must occur in the context of a broader systems change—both within health and outside of health—as outlined in key national policy documents [1, 38]. In other words, general practice obesity prevention efforts should complement other concurrent preventive actions such as upstream policy change [39], as part of a holistic, whole‐of‐society approach [37].

### 6.1 Strengths and Limitations

Strengths of this mapping review include extensive coverage of websites from around Australia, independent assessment in duplicate by two practising GP investigators regarding resource suitability for general practice, and a quality assessment of all patient‐facing materials with the respected PEMAT tool.

In terms of limitations, we did not include password‐protected websites such as Healthpathways, recognised as a source of localised information for primary care practitioners, which may be used during consultations [40]. However, this was done to ensure that resources included in this review were readily available to all potential users. We also acknowledge that the exclusion of websites with video content is a limitation of this review, particularly as some users may prefer video content as an information source. However, for those with limited (or no) access to the internet, accessing web‐based content can be challenging and potentially exacerbates existing health inequities [41]. This is particularly relevant given that many priority populations in Australia, such as children living in the lowest socioeconomic areas or with a disability, are at a relatively higher risk of developing overweight and obesity [42]. Other limitations of the review included a single person screening resources with regards to eligibility criteria, and although we specifically flagged resources for culturally and linguistically diverse groups in this report, materials in languages other than English were excluded.

Finally, as the scope of this review was limited to resources for use in the context of a patient consultation, there is an opportunity for other reviews to examine other types of resources, which would also be relevant to obesity prevention in general practice. Additionally, the development of a validated tool to assess the quality of resources targeted towards GPs would be helpful for similar research in the future.

## 7 Conclusion

Clinical resources have the potential to play an important role in obesity prevention within the context of general practice. This review found many relevant Australian clinical resources. The majority were directed towards children and their families; however, gaps between understandability and actionability were identified in resources suitable for patients. Collaborative work with GPs and families to ensure clinical resources are relevant, readily accessible, and impactful is critical. We recommend that future research focus on working directly with GPs to ensure that they are adequately connected to existing resources and that the resources meet GPs' needs to support obesity prevention in their practice. Partnering with families to understand what guidance is needed to put concepts into action is also critical. Although we recognise that general practice cannot single‐handedly address the high rate of obesity prevalence amongst Australian children, as part of a whole systems approach, GPs have a very important contribution to make.

## Conflicts of Interest

The authors declare no conflicts of interest.

## Supporting information


**Data S1** Supporting Information.

## Data Availability

The data that support the findings of this study are available from the corresponding author upon reasonable request.
