# Supplementary material for: Childhood Obesity Prevention and General Practice: A Mapping Review of Australian Clinical Resources
Source: Health Promot J Austr. 2025 Apr 9;36(2):e70006. doi: 10.1002/hpja.70006 (PMC11979556; doi:10.1002/hpja.70006)
Supplement: Supplementary file 1 — Data S1 Supporting Information. [file HPJA-36-0-s001.pdf]

Supplementary material:

Grey literature search

Direct search of websites

Royal Australian College of General Practitioners (RACGP)	https://www.racgp.org.au/
National Aboriginal Community Controlled Health Organisation (NACCHO)	https://www.naccho.org.au/
Primary Healthcare Networks	https://www.health.gov.au/our-work/phn
Australian Government Department of Health	https://www.health.gov.au/<br><br>https://www.eatforhealth.gov.au
National Health and Medical Research Council (NHMRC)	https://www.nhmrc.gov.au/
Australian State and Territory government Department of Health (or equivalent)	Victoria<br>• https://www.vic.gov.au/department-health<br>• https://prevention.health.vic.gov.au/<br><br>NSW<br>• https://www.health.nsw.gov.au/<br><br>Queensland<br>• https://www.health.qld.gov.au/<br><br>South Australia<br>• https://www.sahealth.sa.gov.au/wps/wcm/connect/public+content/sa+health+internet<br><br>Western Australia<br>• https://ww2.health.wa.gov.au/<br>• https://www.healthywa.wa.gov.au/<br><br>Tasmania<br>• https://www.health.tas.gov.au/<br><br>Northern Territory<br>• https://health.nt.gov.au/<br><br>Australian Capital Territory<br>• https://health.act.gov.au/
Health and Wellbeing Queensland	https://hw.qld.gov.au/
VicHealth	https://www.vichealth.vic.gov.au/
Healthway (WA)	https://www.healthway.wa.gov.au/
Wellbeing SA	https://www.wellbeingsa.sa.gov.au/
Healthy kids for professionals (NSW)	http://pro.healthykids.nsw.gov.au/
Royal Children's Hospital (Vic)	https://www.rch.org.au/home/

Monash children's hospital (Vic)	https://monashchildrenshospital.org/
Sydney Children's Hospital network	https://www.schn.health.nsw.gov.au/hospitals
John Hunter Children's Hospital (NSW)	https://www.hnekidshealth.nsw.gov.au/
Perth children's hospital	https://pch.health.wa.gov.au/
Queensland's children's hospital	https://www.childrens.health.qld.gov.au/qch/
Women's and Children's hospital (SA)	https://www.wch.sa.gov.au/
Centenary Hospital for Women and Children (ACT)	https://www.canberrahealthservices.act.gov.au/home
Raising children network	https://raisingchildren.net.au/

Google scholar search using search terms:

- obesity childhood prevention general practitioner resource
- healthy eating general practitioner resource
- breastfeeding general practitioner children
- healthy sleep general practitioner children
- healthy physical activity general practitioner children
- healthy sedentary activity general practitioner children

The first 40 'hits' of each search were reviewed.

Duck Duck Go using search terms:

- Obesity childhood prevention general practitioner resource
- Healthy eating general practitioner children
- physical activity general practitioner children
- sedentary activity general practitioner children
- sleep general practitioner children
- breastfeeding general practitioner

Search was restricted to Australian websites and the first 40 'hits' of each individual search were reviewed.

Full list of resources included in review

Children/family resources

**Source**	**Title**
Australian Capital Territory (ACT) Government	Kids at play active play everyday
ACT Health	Kids at play switch to play everyday
ACT Health Canberra Hospital and Health Services	Foodwise Drink up
	Foodwise Healthy eating for young people
Better Health Channel	Children - keeping them active
	Eating tips for preschoolers
	Eating tips for school children
	Growth and development - primary school children
	Growth charts for children
	Kids and energy needs
	Soft drinks, juice and sweet drinks - children
	Sport and children
	Teenagers and sleep
Canberra Health services	Tuckatalk drink up for good health
	Tuckatalk baby's first foods
	Tuckatalk food for your child (4-6 years old)
	Tuckatalk food for your toddler (1-3 years old)
	Tuckatalk Healthy lifestyles for healthy bodies
Centre for Children’s Health and Wellbeing, Children’s Health Queensland Hospital and Health Service	Baby’s first foods. From birth to 12 months For mums, dads, families and carers
	Baby's first foods
Children's Health Queensland	MPI team Activity warriors
	MPI Team
Children's health Queensland Hospital and Health service	Physical activity for the family
	Healthy sleep in children
	Good start to life Healthy Kids 1 to 4 years of age
Department of Health and Ageing, Australian Government	Giving your baby the best start
	Healthy eating for children. Teach your child healthy habits for a healthy life
Department of Health, Australian Government	Guidelines for healthy growth and development for children and young people (5 to 17 years)
	Guidelines for healthy growth and development for your child - the early years (brochure)
	Is your family missing out on the benefits of being active every day?
	Have you moved enough today?
Department of Health, Northern Territory	Feeding babies
Department of Health, Queensland	Health guidelines
	Sugary drink facts
	7 dinnertime habits to improve your child’s health
	Serving sizes for kids

Department of Health, Tasmania	Start them right A parent's guide to eating for under 5s
Health and Wellbeing Queensland	Creating healthy teen sleep habits: A guide for parents of teenagers
Healthdirect	Benefits of physical activity for children
	Healthy eating for your children
	How will you feed your baby?
	How your baby gains weight
	Sleep tips for children
Healthy Tasmania	Physical activity for children 0-5 years
	Physical activity for children 5-12 years
	Physical activity for young people 13-17 years
	Sitting and screens for children 5-12 years
	Sitting and screens for young people 13-17 years
	Screen and sitting for children 0-5 years
	Sleep for children 0-5 years
	Active play, sitting and sleep children 0-5 years poster
	Move more and sit less children 5-12 years
	Move more and sit less young people 13-17 years
	Drink mostly water and plain milk
Healthy WA	Food for kids
	Tips for being active
	Toddler Tucker - healthy eating for 1 to 3 year olds
Kids health - The children's hospital at Westmead	Breastfeeding
	Physical activity
Monash Children's Hospital	Healthy Lifestyles for Children and Adolescents
Nutrition Education Materials Online (NEMO)	Healthy eating for Toddlers
NSW Government	Healthy weight calculator for children and teenagers
	We measure the height and weight of all children
NSW Health	Body mass index (BMI)–for-age percentile chart; boys 2-18 years Healthy Eating Active Living
	Body mass index (BMI)–for-age percentile chart; boys 2 to 18 years Healthy kids for professionals
	Body mass index (BMI)–for-age percentile chart; 2 to 18 years Healthy Eating Active Living
	Body mass index (BMI)–for-age percentile chart; 2-18 years<br>Healthy kids for professionals
	8 Healthy Habits 2-17 years
	8 Healthy habits 0-12 months
	8 for a healthy weight
	8 healthy habits 12-24 months

Perth children's hospital	Growth (Keeping our mob healthy)
Queensland Department of Health	Healthy foods and drinks for children aged 1 - 4 years
	Breastfeeding: good for baby, good for mum
	Healthy food and drinks for your grandkids
	Healthy drinks for baby
Queensland Government	Benefits of Breastfeeding
	Exercise, sleep and screentime - what do kids need
	Breastfeeding is awesome
	Food and drinks a growing baby needs
Queensland Health	How children develop: food and nutrition 1 to 5 years
Raising Children Network	Better sleep for pre-teens and teenagers
	Dietary guidelines children 1-2 years
	Dietary guidelines children 2-3 years
	Dietary guidelines children 4-8 years
	Child growth charts
	Healthy drinks for kids and teenagers
	Healthy food for school age children: the five food groups
	Healthy food for babies and toddlers: the five food groups
	Healthy food every day
	Nutrition and healthy food for teenagers
	Physical activity for babies and children: why and how much
	Physical activity for school age children
	Physical activity for young children
	Physical activity for pre-teens and teenagers
	About sleep
Royal Children's Hospital	Is my child growing well? Questions and answers for parents
	Breastfeeding (Kids Health Information)
SA Health	Fitness and exercise for kids and teens
	Healthy eating at home
	Healthy eating for babies and young children
	Healthy eating for kids and teens
	Physical activity for babies, toddlers and pre-schoolers
Sydney Children's Hospital Network	Healthy eating for toddlers
	How to get kids to be more active
	Normal sleeping patterns 0-16 years
Tasmanian Department of Health	Breastfeeding the natural way to feed your baby
	Drinks for Children Make water the main drink
	First foods for your baby
	Food for under Fives
	Healthy family cooking for growing families
The Children's Hospital at Westmead	Be active for at least one hour a day

RACGP	Join in the fun at parkrun
	We are a parkrun practice

GP resources

Source	Title
Department of Health, Australian Government	Guidelines for healthy growth and development for your child the early years (birth to 5 years)
	Australian 24-hour movement guidelines for children and young people (5 to 17 years)
NSW Government	Guide to accurately measuring the height of a child
	Guide to accurately measuring the length of a child
	Guide to accurately weighing children using standing scales
	Guide to accurately weighing children using infant scales

The Patient Education Materials Assessment Tool (PEMAT)*

PEMAT-P for Printable Materials (PEMAT-P):

Understandability

1. The material makes its purpose completely evident (Disagree=0, Agree=1)

2. The material does not include information or content that distracts from its purpose (Disagree=0, Agree=1)

3. The material uses common, everyday language (Disagree=0, Agree=1)

4. Medical terms are used only to familiarize audience with the terms. When used, medical terms are defined language (Disagree=0, Agree=1)

5. The material uses the active voice language (Disagree=0, Agree=1)

6. Numbers appearing in the material are clear and easy to understand (Disagree=0, Agree=1, No numbers=N/A)

7. The material does not expect the user to perform calculations (Disagree=0, Agree=1)

8. The material breaks or "chunks" information into short sections (Disagree=0, Agree=1, Very short material=N/A)

9. The material's sections have informative headers (Disagree=0, Agree=1, Very short material=N/A)

10. The material presents information in a logical sequence (Disagree=0, Agree=1)

11. The material provides a summary (Disagree=0, Agree=1, Very short material=N/A)

12. The material uses visual cues (e.g., arrows, boxes, bullets, bold, larger font, highlighting) to draw attention to key points. (Disagree=0, Agree=1)

15. The material uses visual aids whenever they could make content more easily understood (e.g., illustration of healthy portion size) (Disagree=0, Agree=1)

16. The material’s visual aids reinforce rather than distract from the content (Disagree=0, Agree=1, No visual aids=N/A)

17. The material’s visual aids have clear titles or captions (Disagree=0, Agree=1, No visual aids=N/A)

18. The material uses illustrations and photographs that are clear and uncluttered (Disagree=0, Agree=1, No visual aids=N/A)

19. The material uses simple tables with short and clear row and column headings (Disagree=0, Agree=1, No tables=N/A)

Understandability Score (%) = Total Points / Total Possible Points x 100

Actionability

20. The material clearly identifies at least one action the user can take. (Disagree=0, Agree=1)

21. The material addresses the user directly when describing actions. (Disagree=0, Agree=1)

22. The material breaks down any action into manageable, explicit steps. (Disagree=0, Agree=1)

23. The material provides a tangible tool (e.g., menu planners, checklists) whenever it could help the user take action. (Disagree=0, Agree=1)

24. The material provides simple instructions or examples of how to perform calculations. (Disagree=0, Agree=1, No calculations=N/A)

25. The material explains how to use the charts, graphs, tables, or diagrams to take actions (Disagree=0, Agree=1, No charts, graphs, tables, or diagrams=N/A)

26. The material uses visual aids whenever they could make it easier to act on the instructions (Disagree=0, Agree=1)

Actionability Score (%) = Total Points / Total Possible Points x 100

<u>PEMAT Tool for Audiovisual Materials (PEMAT - A/V):</u>

Understandability

1. The material makes its purpose completely evident (Disagree=0, Agree=1)

3. The material uses common, everyday language (Disagree=0, Agree=1)

4. Medical terms are used only to familiarize audience with the terms. When used, medical terms are defined language (Disagree=0, Agree=1)

5. The material uses the active voice language (Disagree=0, Agree=1)

8. The material breaks or "chunks" information into short sections (Disagree=0, Agree=1, Very short material=N/A)

9. The material's sections have informative headers (Disagree=0, Agree=1, Very short material=N/A)

10. The material presents information in a logical sequence (Disagree=0, Agree=1)

11. The material provides a summary (Disagree=0, Agree=1, Very short material=N/A)

12. The material uses visual cues (e.g., arrows, boxes, bullets, bold, larger font, highlighting) to draw attention to key points. (Disagree=0, Agree=1)

13. Text on the screen is easy to read (Disagree=0, Agree=1, No text or all text is narrated=N/A)

14. The material allows the user to hear the words clearly (e.g., not too fast, not garbled) (Disagree=0, Agree=1, No narration=N/A

18. The material uses illustrations and photographs that are clear and uncluttered (Disagree=0, Agree=1, No visual aids=N/A)

19. The material uses simple tables with short and clear row and column headings (Disagree=0, Agree=1, No tables=N/A)

Understandability Score (%) = Total Points / Total Possible Points x 100

Actionability

20. The material clearly identifies at least one action the user can take. (Disagree=0, Agree=1)

21. The material addresses the user directly when describing actions. (Disagree=0, Agree=1)

22. The material breaks down any action into manageable, explicit steps. (Disagree=0, Agree=1)

25. The material explains how to use the charts, graphs, tables, or diagrams to take actions (Disagree=0, Agree=1, No charts, graphs, tables, or diagrams=N/A)

Actionability Score (%) = Total Points / Total Possible Points x 100

*Reference: The Patient Education Materials Assessment Tool (PEMAT) and User's Guide (Version 1.0) available at https://www.ahrq.gov/health-literacy/patient-education/pemat-p.html; accessed 1 December 2023

Quality assessment tool for GP resources (adapted from the RACGP guideline assessment tool[#^])

1. The resource is clearly written in an understandable form and the key recommendations are easily identifiable.

2. The target users and the health care context addressed by the resource explicitly include general practitioners

3. The background and aims of the guidance/resource as well as the patients to whom the resource is meant to apply are clearly described.

4. The members of the resource development group have stated their conflicts of interest and the financial independence has documented and transparently managed.

5. An indication that the resource is likely to be evidence based eg. provision of references, link to further information

6. Clear designation about the currency of the resource and/or date of the last search are provided.

[#]Adapted from the RACGP guideline assessment tool^:

- *Question 1, 6, 7 and 10 from original tool are excluded as they are either outside the scope of the review or are not applicable to the clinical resources we are seeking or the content area.*
- *Question 5 from original tool – requirement has been simplified*
- *Reference to "guidelines" has been changed to "resources" or deleted as applicable*
- *Added scoring (Yes = 1, No = 0) and removed option of "partly"*

^The RACGP guideline assessment tool is available at https://www.racgp.org.au/FSDEDEV/media/documents/Clinical%20Resources/Resources/RACGP-guideline-assessment-tool.pdf; accessed 1 December 2023
